# Crystal structure of (*E*)-2-[(2*S*,5*R*)-2-isopropyl-5-methyl­cyclo­hexyl­idene]hydrazine-1-carbo­thio­amide

**DOI:** 10.1107/S1600536814015980

**Published:** 2014-08-01

**Authors:** Adriano Bof de Oliveira, Johannes Beck, Jörg Daniels, Renan Lira de Farias, Adelino Vieira de Godoy Netto

**Affiliations:** a Departamento de Química, Universidade Federal de Sergipe, Av. Marechal Rondon s/n, Campus, 49100-000 São Cristóvão-SE, Brazil; b Institut für Anorganische Chemie, Universität Bonn, Gerhard-Domagk-Strasse 1, D-53121 Bonn, Germany; c Instituto de Química, Universidade Estadual Paulista, Rua Francisco Degni s/n, 14801-970 Araraquara-SP, Brazil

**Keywords:** thio­carbazone, hydrogen-bonding polymer, crystal structure

## Abstract

The title compound, C_11_H_21_N_3_S, consists of a menthone moiety attached to an extended thio­semicarbazone group with the N—N—C—N torsion angle being 11.92 (16)°. The cyclo­hexane ring has a chair conformation and the conformation about the C=N bond is *E*. In the crystal, mol­ecules are linked *via* pairs of N—H⋯S hydrogen bonds, forming chains along the *a* axis. The absolute structure could be assigned with reference to the starting material, *i.e.* enanti­opure (−)-menthone [Flack parameter = 0.05 (5)].

## Related literature   

For one of the first reports of the synthesis of thio­semicarbazone derivatives, see: Freund & Schander (1902 ▶). For a report of the anti-HIV activity of thio­semicarbazone derivatives of menthone, see: Mishra *et al.* (2012 ▶).
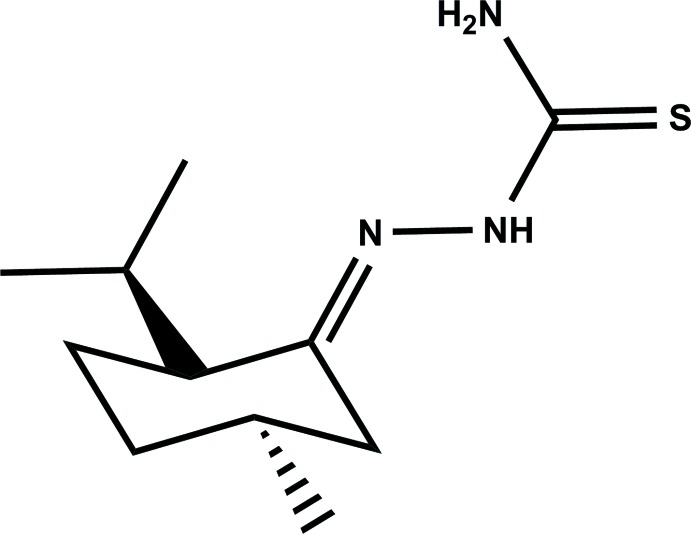


## Experimental   

### Crystal data  

C_11_H_21_N_3_S
*M* *_r_* = 227.37
Orthorhombic, 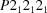

*a* = 8.2139 (1) Å
*b* = 11.6117 (2) Å
*c* = 13.8820 (2) Å
*V* = 1324.03 (3) Å^3^
*Z* = 4
Mo *K*α radiation
μ = 0.22 mm^−1^
*T* = 123 K
0.54 × 0.10 × 0.06 mm

### Data collection  

Nonius KappaCCD diffractometer
Absorption correction: analytical (Alcock, 1970 ▶) *T*_min_ = 0.890, *T*_max_ = 0.988
22998 measured reflections
3033 independent reflections
2848 reflections with *I* > 2σ(*I*)
*R*_int_ = 0.046

### Refinement  

*R*[*F* ^2^ > 2σ(*F* ^2^)] = 0.025
*wR*(*F* ^2^) = 0.060
*S* = 1.05
3033 reflections
220 parameters
All H-atom parameters refined
Δρ_max_ = 0.15 e Å^−3^
Δρ_min_ = −0.19 e Å^−3^
Absolute structure: Flack (1983 ▶)
Absolute structure parameter: 0.05 (5)

### 

Data collection: *COLLECT* (Nonius, 1998 ▶); cell refinement: *SCALEPACK* (Otwinowski & Minor, 1997 ▶); data reduction: *DENZO* (Otwinowski & Minor, 1997 ▶) and *SCALEPACK*; program(s) used to solve structure: *SHELXS97* (Sheldrick, 2008 ▶); program(s) used to refine structure: *SHELXL97* (Sheldrick, 2008 ▶); molecular graphics: *DIAMOND* (Brandenburg, 2006 ▶); software used to prepare material for publication: *publCIF* (Westrip, 2010 ▶).

## Supplementary Material

Crystal structure: contains datablock(s) I, publication_text. DOI: 10.1107/S1600536814015980/su2751sup1.cif

Structure factors: contains datablock(s) I. DOI: 10.1107/S1600536814015980/su2751Isup2.hkl

Supporting information file. DOI: 10.1107/S1600536814015980/su2751Isup3.cml

**Fig. 1.**
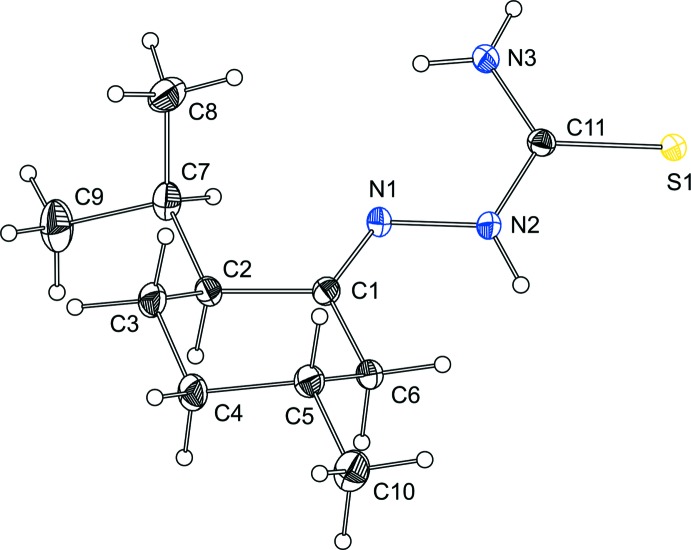
The molecular structure of the title molecule, with atom labelling. Displacement ellipsoids are drawn at the 40% probability level.

**Fig. 2.**
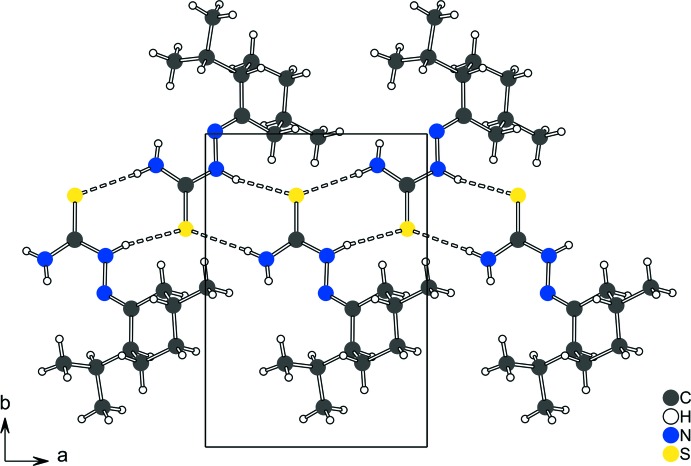
A partial view along the c-axis of the crystal structure of the title compound, showing the hydrogen bonded chains (hydrogen bonds are shown as dashed lines; see Table 1 for details).

. DOI: 10.1107/S1600536814015980/su2751fig1.tif
The mol­ecular structure of the title mol­ecule, with atom labelling. Displacement ellipsoids are drawn at the 40% probability level.

c . DOI: 10.1107/S1600536814015980/su2751fig2.tif
A partial view along the *c*-axis of the crystal structure of the title compound, showing the hydrogen bonded chains (hydrogen bonds are shown as dashed lines; see Table 1 for details).

CCDC reference: 1012829

Additional supporting information:  crystallographic information; 3D view; checkCIF report

## Figures and Tables

**Table 1:** Hydrogen-bond geometry (Å, °)

*D*—H⋯*A*	*D*—H	H⋯*A*	*D*⋯*A*	*D*—H⋯*A*
N2—H*N*2⋯S1^i^	0.858 (17)	2.525 (18)	3.3551 (11)	163.1 (15)
N3—H*N*3*A*⋯S1^ii^	0.794 (19)	2.52 (2)	3.3104 (12)	173.0 (17)

Symmetry codes: (i) 
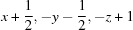
; (ii) 
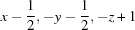
.

## supplementary crystallographic information

### S1. Structural commentary

Several thio­semicarbazone derivatives have a wide range of pharmacological properties. For example, some thio­semicarbazone derivatives of the peppermint essential oil show anti-HIV activity (Mishra *et al.*, 2012). As part of our studies on synthesis and structural chemistry of thio­semicarbazone derivatives of natural products, we report herein the crystal structure of (-)-3-menthone thio­semicarbazone.

In the molecular structure of the title compound, Fig, 1, the thio­semicarbazone unit is not completely planar, but shows a torsion angle N1–N2–C11–N3 of 11.92 (16)°. The cyclo­hexane ring of the menthone unit is in the chair conformation. The molecule, shows also a *trans* conformation about the N1—N2 bond.

For the synthesis, enanti­opure (-)-menthone was used. No change in chirality occurred in the course of the reaction with thio­semicarbazide and the obtained product emerged as chiral crystals in the non-centrosymmetric space group *P*2_1_2_1_2_1_.

In the crystal, molecules are connected by a pair of N—H···S hydrogen bonds, with bridging sulfur atoms, into a one-dimensional chain along the *a*-axis (Fig. 2 and Table 1).

### S2. Synthesis and crystallization

The synthesis of the title compound was adapted from a previously reported procedure (Freund & Schander, 1902). In a hydro­chloric acid catalyzed reaction, a mixture of (-)-menthone (10 mmol) and thio­semicarbazide (10 mmol) in ethanol (80 ml) was refluxed for 5 h. After cooling and filtering, the title compound was obtained. Colourless needles were obtained by slow evaporation of a solution in the solvent DMSO.

### S3. Refinement

All the H atoms were located in a difference Fourier map and freely refined. The assignment of the correct absolute configuration was assured by the Flack parameter of 0.05 (5).

### Crystal data

**Table:**

C_11_H_21_N_3_S	*F*(000) = 496
*M**_r_* = 227.37	*D*_x_ = 1.141 Mg m^−^^3^
Orthorhombic, *P*2_1_2_1_2_1_	Mo *K*α radiation, λ = 0.71073 Å
Hall symbol: P 2ac 2ab	Cell parameters from 37558 reflections
*a* = 8.2139 (1) Å	θ = 2.9–27.5°
*b* = 11.6117 (2) Å	µ = 0.22 mm^−^^1^
*c* = 13.8820 (2) Å	*T* = 123 K
*V* = 1324.03 (3) Å^3^	Needle, colourless
*Z* = 4	0.54 × 0.10 × 0.06 mm

### Data collection

**Table:**

Nonius KappaCCD diffractometer	3033 independent reflections
Radiation source: fine-focus sealed tube, Nonius KappaCCD	2848 reflections with *I* > 2σ(*I*)
Graphite monochromator	*R*_int_ = 0.046
Detector resolution: 9 pixels mm^-1^	θ_max_ = 27.5°, θ_min_ = 2.9°
CCD rotation images, thick slices scans	*h* = −10→10
Absorption correction: analytical (Alcock, 1970)	*k* = −15→15
*T*_min_ = 0.890, *T*_max_ = 0.988	*l* = −17→18
22998 measured reflections	

### Refinement

**Table:**

Refinement on *F*^2^	Secondary atom site location: difference Fourier map
Least-squares matrix: full	Hydrogen site location: inferred from neighbouring sites
*R*[*F*^2^ > 2σ(*F*^2^)] = 0.025	All H-atom parameters refined
*wR*(*F*^2^) = 0.060	*w* = 1/[σ^2^(*F*_o_^2^) + (0.0305*P*)^2^ + 0.1951*P*] where *P* = (*F*_o_^2^ + 2*F*_c_^2^)/3
*S* = 1.05	(Δ/σ)_max_ = 0.001
3033 reflections	Δρ_max_ = 0.15 e Å^−^^3^
220 parameters	Δρ_min_ = −0.19 e Å^−^^3^
0 restraints	Absolute structure: Flack (1983), ???? Friedel pairs
Primary atom site location: structure-invariant direct methods	Absolute structure parameter: 0.05 (5)

### Special details

**Table:**

Experimental. Alcock, N. W., 1970
Geometry. All e.s.d.'s (except the e.s.d. in the dihedral angle between two l.s. planes) are estimated using the full covariance matrix. The cell e.s.d.'s are taken into account individually in the estimation of e.s.d.'s in distances, angles and torsion angles; correlations between e.s.d.'s in cell parameters are only used when they are defined by crystal symmetry. An approximate (isotropic) treatment of cell e.s.d.'s is used for estimating e.s.d.'s involving l.s. planes.
Refinement. Refinement of *F*^2^ against ALL reflections. The weighted *R*-factor *wR* and goodness of fit *S* are based on *F*^2^, conventional *R*-factors *R* are based on *F*, with *F* set to zero for negative *F*^2^. The threshold expression of *F*^2^ > σ(*F*^2^) is used only for calculating *R*-factors(gt) *etc*. and is not relevant to the choice of reflections for refinement. *R*-factors based on *F*^2^ are statistically about twice as large as those based on *F*, and *R*- factors based on ALL data will be even larger.

### Fractional atomic coordinates and isotropic or equivalent isotropic displacement parameters (Å^2^)

**Table:**

	*x*	*y*	*z*	*U*_iso_*/*U*_eq_	
S1	−0.08952 (3)	−0.30315 (2)	0.48771 (2)	0.02034 (8)	
N1	0.04374 (12)	0.00856 (9)	0.41416 (7)	0.0177 (2)	
N2	0.04891 (13)	−0.11130 (9)	0.42555 (8)	0.0186 (2)	
N3	−0.22424 (13)	−0.09979 (10)	0.45259 (9)	0.0231 (2)	
C1	0.16488 (14)	0.05821 (10)	0.37313 (8)	0.0168 (2)	
C2	0.16273 (15)	0.18913 (11)	0.36955 (8)	0.0192 (2)	
C3	0.20800 (17)	0.23095 (11)	0.26796 (10)	0.0238 (3)	
C4	0.36682 (16)	0.17718 (11)	0.23241 (11)	0.0263 (3)	
C5	0.35351 (16)	0.04665 (11)	0.22932 (9)	0.0217 (3)	
C6	0.31196 (15)	−0.00025 (11)	0.32996 (9)	0.0193 (2)	
C7	0.00597 (17)	0.24498 (11)	0.40902 (10)	0.0244 (3)	
C8	−0.14038 (18)	0.23265 (14)	0.34230 (13)	0.0337 (3)	
C9	0.0343 (3)	0.37222 (13)	0.43286 (15)	0.0406 (4)	
C10	0.50872 (19)	−0.00988 (14)	0.19164 (12)	0.0320 (3)	
C11	−0.09004 (15)	−0.16286 (9)	0.45455 (8)	0.0166 (2)	
HN2	0.139 (2)	−0.1453 (14)	0.4388 (12)	0.030 (4)*	
HN3A	−0.308 (2)	−0.1263 (15)	0.4708 (13)	0.038 (5)*	
HN3B	−0.214 (2)	−0.0304 (16)	0.4401 (12)	0.032 (4)*	
H2	0.2510 (19)	0.2111 (14)	0.4104 (11)	0.025 (4)*	
H3A	0.1221 (19)	0.2081 (13)	0.2244 (11)	0.026 (4)*	
H3B	0.2167 (19)	0.3172 (14)	0.2688 (11)	0.026 (4)*	
H4A	0.395 (2)	0.2061 (14)	0.1673 (12)	0.038 (4)*	
H4B	0.4556 (19)	0.1942 (14)	0.2754 (11)	0.027 (4)*	
H5	0.2651 (19)	0.0275 (13)	0.1881 (11)	0.022 (4)*	
H6A	0.2988 (18)	−0.0818 (14)	0.3277 (11)	0.024 (4)*	
H6B	0.4050 (19)	0.0163 (12)	0.3714 (10)	0.021 (3)*	
H7	−0.0193 (19)	0.2058 (13)	0.4704 (11)	0.032 (4)*	
H8A	−0.124 (2)	0.2778 (16)	0.2830 (13)	0.046 (5)*	
H8B	−0.237 (2)	0.2586 (16)	0.3744 (12)	0.040 (5)*	
H8C	−0.160 (2)	0.1543 (16)	0.3249 (12)	0.033 (4)*	
H9A	−0.058 (2)	0.4100 (15)	0.4585 (12)	0.037 (5)*	
H9B	0.123 (3)	0.3810 (18)	0.4819 (17)	0.067 (6)*	
H9C	0.063 (2)	0.4138 (15)	0.3757 (14)	0.045 (5)*	
H10A	0.606 (2)	0.0073 (14)	0.2334 (12)	0.039 (4)*	
H10B	0.494 (2)	−0.0917 (18)	0.1884 (13)	0.041 (5)*	
H10C	0.533 (2)	0.0126 (17)	0.1271 (15)	0.053 (6)*	

### Atomic displacement parameters (Å^2^)

**Table:**

	*U*^11^	*U*^22^	*U*^33^	*U*^12^	*U*^13^	*U*^23^
S1	0.01461 (12)	0.01597 (13)	0.03044 (16)	−0.00151 (11)	−0.00087 (12)	0.00710 (12)
N1	0.0187 (5)	0.0138 (5)	0.0206 (5)	−0.0008 (4)	−0.0011 (4)	0.0037 (4)
N2	0.0155 (5)	0.0140 (5)	0.0264 (6)	−0.0001 (4)	0.0001 (4)	0.0059 (4)
N3	0.0150 (5)	0.0163 (5)	0.0380 (7)	−0.0007 (4)	0.0031 (5)	0.0053 (5)
C1	0.0183 (5)	0.0171 (6)	0.0151 (5)	−0.0002 (5)	−0.0028 (5)	0.0026 (4)
C2	0.0201 (5)	0.0157 (5)	0.0217 (6)	−0.0026 (5)	−0.0036 (5)	0.0016 (5)
C3	0.0251 (6)	0.0191 (6)	0.0272 (6)	0.0005 (5)	0.0023 (6)	0.0079 (5)
C4	0.0263 (7)	0.0213 (7)	0.0314 (7)	−0.0008 (5)	0.0073 (6)	0.0095 (5)
C5	0.0219 (6)	0.0219 (6)	0.0213 (6)	−0.0003 (5)	0.0018 (5)	0.0048 (5)
C6	0.0183 (5)	0.0174 (6)	0.0223 (6)	−0.0014 (5)	0.0000 (5)	0.0053 (5)
C7	0.0301 (7)	0.0171 (6)	0.0259 (7)	0.0015 (5)	0.0053 (6)	0.0013 (5)
C8	0.0249 (7)	0.0326 (8)	0.0435 (9)	0.0071 (6)	0.0017 (6)	0.0066 (7)
C9	0.0557 (11)	0.0205 (7)	0.0455 (10)	0.0015 (7)	0.0114 (8)	−0.0055 (7)
C10	0.0324 (7)	0.0295 (8)	0.0342 (8)	0.0045 (6)	0.0108 (7)	0.0073 (6)
C11	0.0159 (5)	0.0174 (5)	0.0165 (5)	−0.0012 (5)	−0.0010 (5)	0.0007 (4)

### Geometric parameters (Å, º)

**Table:**

S1—C11	1.6928 (11)	C4—H4B	0.963 (16)
N1—C1	1.2833 (15)	C5—C10	1.5264 (19)
N1—N2	1.4015 (14)	C5—C6	1.5377 (17)
N2—C11	1.3502 (15)	C5—H5	0.951 (15)
N2—HN2	0.858 (17)	C6—H6A	0.954 (16)
N3—C11	1.3237 (16)	C6—H6B	0.976 (15)
N3—HN3A	0.794 (19)	C7—C8	1.524 (2)
N3—HN3B	0.829 (18)	C7—C9	1.5318 (19)
C1—C6	1.5098 (17)	C7—H7	0.988 (16)
C1—C2	1.5211 (16)	C8—H8A	0.984 (19)
C2—C3	1.5372 (17)	C8—H8B	0.961 (19)
C2—C7	1.5423 (18)	C8—H8C	0.955 (18)
C2—H2	0.955 (15)	C9—H9A	0.947 (18)
C3—C4	1.5282 (18)	C9—H9B	1.00 (2)
C3—H3A	0.966 (16)	C9—H9C	0.96 (2)
C3—H3B	1.004 (16)	C10—H10A	1.008 (19)
C4—C5	1.5202 (17)	C10—H10B	0.96 (2)
C4—H4A	0.991 (16)	C10—H10C	0.95 (2)
			
C1—N1—N2	118.21 (10)	C1—C6—C5	112.27 (10)
C11—N2—N1	116.64 (10)	C1—C6—H6A	111.6 (9)
C11—N2—HN2	117.4 (11)	C5—C6—H6A	110.3 (9)
N1—N2—HN2	120.5 (11)	C1—C6—H6B	107.7 (8)
C11—N3—HN3A	120.0 (13)	C5—C6—H6B	107.0 (8)
C11—N3—HN3B	117.0 (12)	H6A—C6—H6B	107.6 (12)
HN3A—N3—HN3B	122.3 (17)	C8—C7—C9	109.98 (13)
N1—C1—C6	126.49 (10)	C8—C7—C2	113.76 (11)
N1—C1—C2	117.03 (11)	C9—C7—C2	110.83 (12)
C6—C1—C2	116.47 (10)	C8—C7—H7	108.4 (9)
C1—C2—C3	110.06 (10)	C9—C7—H7	106.8 (9)
C1—C2—C7	114.73 (10)	C2—C7—H7	106.7 (9)
C3—C2—C7	113.27 (10)	C7—C8—H8A	110.6 (11)
C1—C2—H2	103.8 (10)	C7—C8—H8B	110.0 (10)
C3—C2—H2	106.1 (9)	H8A—C8—H8B	109.4 (16)
C7—C2—H2	108.1 (9)	C7—C8—H8C	112.0 (10)
C4—C3—C2	111.94 (11)	H8A—C8—H8C	108.5 (15)
C4—C3—H3A	108.0 (9)	H8B—C8—H8C	106.2 (15)
C2—C3—H3A	108.1 (9)	C7—C9—H9A	113.9 (10)
C4—C3—H3B	110.5 (9)	C7—C9—H9B	110.8 (12)
C2—C3—H3B	108.8 (9)	H9A—C9—H9B	106.3 (15)
H3A—C3—H3B	109.4 (12)	C7—C9—H9C	110.1 (10)
C5—C4—C3	110.80 (10)	H9A—C9—H9C	106.0 (15)
C5—C4—H4A	109.2 (9)	H9B—C9—H9C	109.5 (16)
C3—C4—H4A	110.8 (10)	C5—C10—H10A	112.4 (10)
C5—C4—H4B	106.1 (10)	C5—C10—H10B	109.9 (11)
C3—C4—H4B	111.2 (9)	H10A—C10—H10B	108.7 (15)
H4A—C4—H4B	108.6 (13)	C5—C10—H10C	112.4 (12)
C4—C5—C10	112.23 (11)	H10A—C10—H10C	108.5 (15)
C4—C5—C6	110.09 (10)	H10B—C10—H10C	104.6 (16)
C10—C5—C6	110.15 (11)	N3—C11—N2	116.90 (10)
C4—C5—H5	107.7 (9)	N3—C11—S1	122.69 (9)
C10—C5—H5	109.3 (9)	N2—C11—S1	120.37 (9)
C6—C5—H5	107.2 (9)		
			
C1—N1—N2—C11	−169.70 (11)	C3—C4—C5—C6	−58.39 (15)
N2—N1—C1—C6	3.59 (18)	N1—C1—C6—C5	133.17 (12)
N2—N1—C1—C2	−174.83 (9)	C2—C1—C6—C5	−48.41 (14)
N1—C1—C2—C3	−133.96 (11)	C4—C5—C6—C1	52.25 (14)
C6—C1—C2—C3	47.46 (14)	C10—C5—C6—C1	176.53 (11)
N1—C1—C2—C7	−4.82 (15)	C1—C2—C7—C8	−73.69 (14)
C6—C1—C2—C7	176.60 (11)	C3—C2—C7—C8	53.83 (15)
C1—C2—C3—C4	−52.04 (14)	C1—C2—C7—C9	161.77 (12)
C7—C2—C3—C4	178.03 (11)	C3—C2—C7—C9	−70.71 (15)
C2—C3—C4—C5	59.56 (15)	N1—N2—C11—N3	11.92 (16)
C3—C4—C5—C10	178.54 (12)	N1—N2—C11—S1	−170.14 (8)

### Hydrogen-bond geometry (Å, º)

**Table:**

*D*—H···*A*	*D*—H	H···*A*	*D*···*A*	*D*—H···*A*
N2—H*N*2···S1^i^	0.858 (17)	2.525 (18)	3.3551 (11)	163.1 (15)
N3—H*N*3*A*···S1^ii^	0.794 (19)	2.52 (2)	3.3104 (12)	173.0 (17)

Symmetry codes: (i) *x*+1/2, −*y*−1/2, −*z*+1; (ii) *x*−1/2, −*y*−1/2, −*z*+1.

## References

[bb1] Alcock, N. W. (1970). *Crystallographic Computing*, edited by F. R. Ahmed, S. R. Hall & C. P. Huber, p. 271. Copenhagen: Munksgaard.

[bb2] Brandenburg, K. (2006). *DIAMOND* Crystal Impact GbR, Bonn, Germany.

[bb3] Flack, H. D. (1983). *Acta Cryst.* A**39**, 876–881.

[bb4] Freund, M. & Schander, A. (1902). *Chem. Ber.* **35**, 2602–2606.

[bb5] Mishra, V., Pandeya, S. N., Pannecouque, C., Witvrouw, M. & De Clercq, E. (2012). *Arch. Pharm. Pharm. Med. Chem.* **5**, 183–186. 10.1002/1521-4184(200205)335:5<183::AID-ARDP183>3.0.CO;2-U 12210774

[bb6] Nonius (1998). *COLLECT* Nonius BV, Delft, The Netherlands.

[bb7] Otwinowski, Z. & Minor, W. (1997). *Methods in Enzymology, Vol. 276, Macromolecular Crystallography, Part A*, edited by C. W. Carter Jr & R. M. Sweet, pp. 307–326. New York: Academic Press, United States.

[bb8] Sheldrick, G. M. (2008). *Acta Cryst.* A**64**, 112–122. 10.1107/S0108767307043930 18156677

[bb9] Westrip, S. P. (2010). *J. Appl. Cryst.* **43**, 920–925.

