# Supplementary figures and images for: Crystal structure of (E)-2-[(2S,5R)-2-isopropyl-5-methyl­cyclo­hexyl­idene]hydrazine-1-carbo­thio­amide
Source: Acta Crystallogr Sect E Struct Rep Online. 2014 Aug 1;70(Pt 9):o903–4. doi: 10.1107/S1600536814015980 (PMC4186105; doi:10.1107/S1600536814015980)

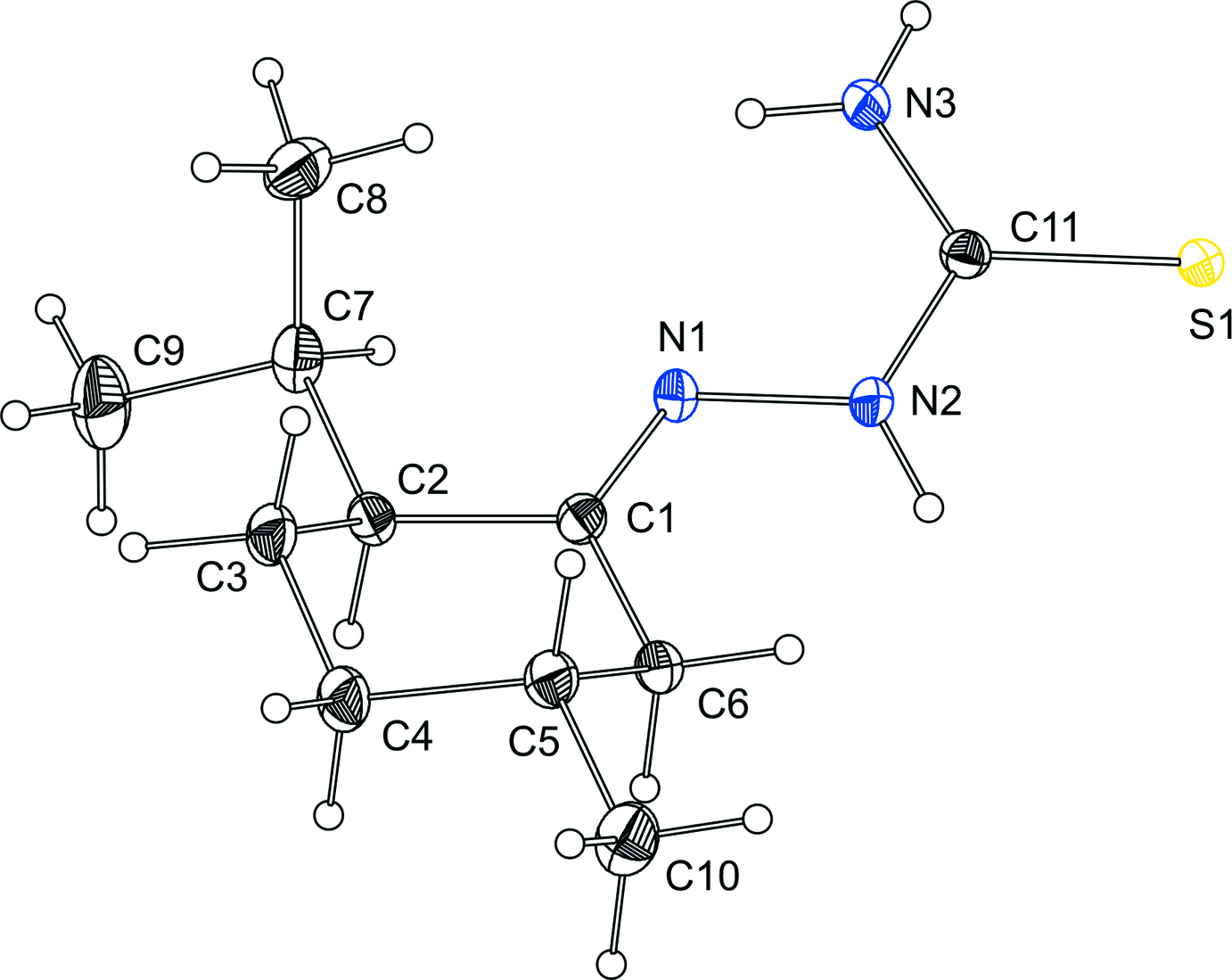

Supplement: Supplementary file 4 [file e-70-0o903-fig1.tif]

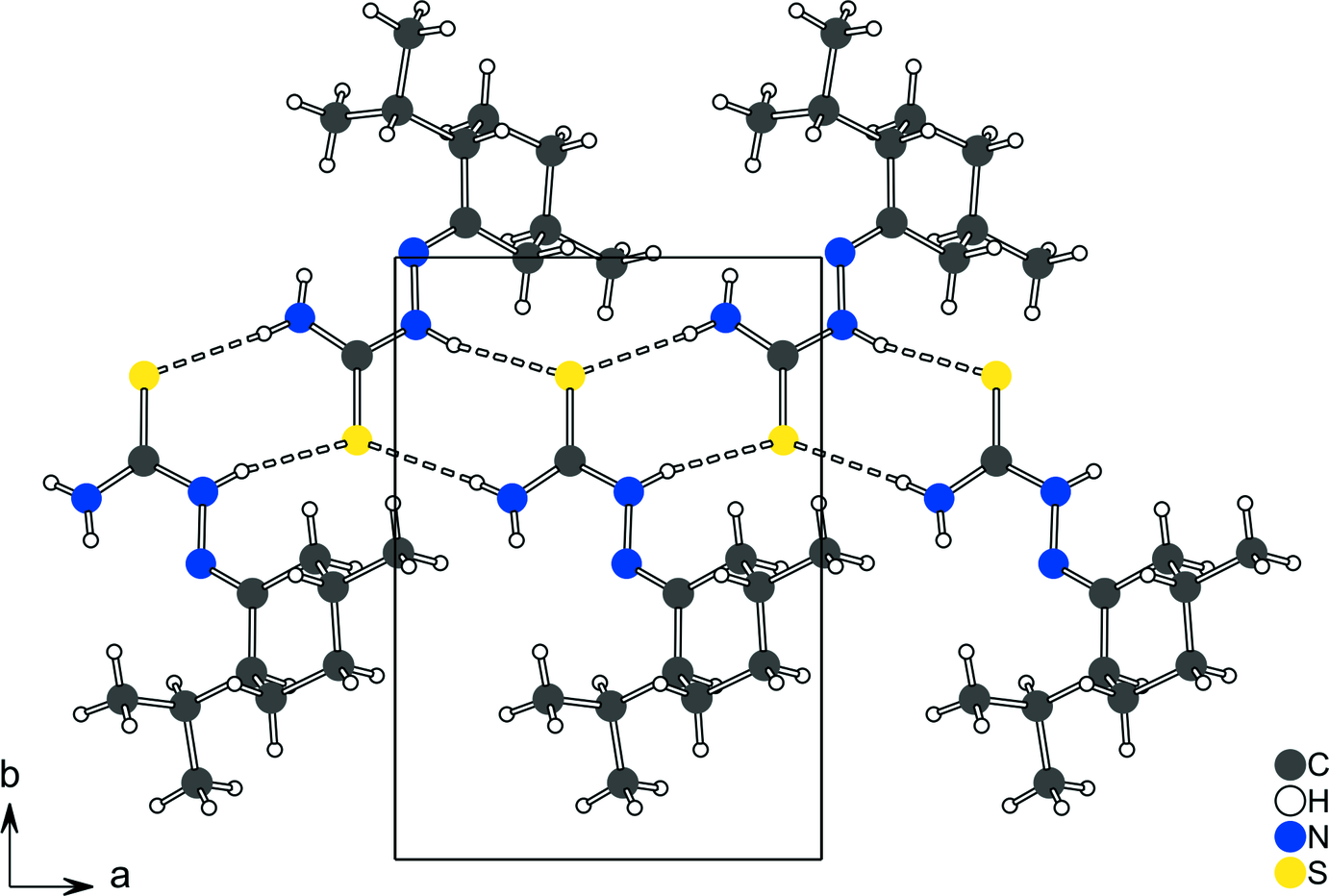

Supplement: Supplementary file 5 [file e-70-0o903-fig2.tif]
